# Mental health disparities between Roma and non-Roma children in Romania and Bulgaria

**DOI:** 10.1186/s12888-014-0297-5

**Published:** 2014-11-18

**Authors:** Eric J Lee, Katherine Keyes, Adina Bitfoi, Zlatka Mihova, Ondine Pez, Elisha Yoon, Viviane Kovess Masfety

**Affiliations:** Department of Epidemiology, Columbia University, New York, New York USA; EHESP Rennes, Sorbonne Paris City, EA 4069 University Paris Descartes, Paris, France; The Romanian League for Mental Health, Bucharest, Romania; New Bulgarian University, Sofia, Bulgaria

**Keywords:** Roma, Mental Health, Romania, Bulgaria, Europe

## Abstract

**Background:**

The Roma population, one of the largest minority groups in Europe, experience discrimination and stigma associated with marginalized social position. Few studies have examined mental illnesses in the Roma, and none have examined the Roma children. The present study estimates mental health and behavioral disorders among Roma children in comparison to non-Roma children in educational institutions.

**Methods:**

Data were drawn from the School Children Mental Health Study in Europe (SCHME) study in Romania (Roma children identified by parent report, N = 70; non-Roma, N = 925) and Bulgaria (Roma children identified by exclusively-Roma schools, N = 65; non-Roma, N = 1312). The Strengths and Difficulties Questionnaire was given to the parents and teachers to measure child mental health; children reported on their mental health through the Dominique Interactive. Control covariates included child sex and age, and parental characteristics when parent reports were available.

**Results:**

Based on the child’s own report, Roma children had a higher odds of any internalizing disorder (OR = 2.99, 95% C.I. 2.07–4.30), phobias (OR = 4.84, 95% C.I. 3.19–7.35), separation anxiety disorder (OR = 2.54, 95% C.I. 1.72–3.76), generalized anxiety disorder (OR = 2.95, 95% C.I. 1.75–4.96), and major depressive disorder (OR = 3.86, 95% C.I. 2.31–6.37). Further Roma children had a higher odds of any externalizing disorder (OR = 2.84, 95% C.I. 1.78–4.54), oppositional defiant disorder (OR = 3.35, 95% C.I. 1.93–5.82), ADHD (OR = 2.37, 95% C.I. 1.26–4.46), and conduct disorder (OR = 3.63, 95% C.I. 2.04–6.46). Based on the report of teachers, Roma children had higher odds of emotional problems (OR = 2.03, 95% C.I. 1.20-3.44), peer-relational problems (OR = 2.76, 95% C.I. 1.73-4.41) and prosocial behavior (OR = 2.75, 95% C.I. 1.75-4.33).

**Conclusion:**

Roma children experience a higher burden of mental health problems compared with their non-Roma counterparts. Attention to child health and mental health among the Roma is urgently needed, as these children experience a constellation of health problems associated with poverty as well as experiences of stigma and discrimination.

**Electronic supplementary material:**

The online version of this article (doi:10.1186/s12888-014-0297-5) contains supplementary material, which is available to authorized users.

## Background

The Roma population is a diverse minority group traditionally known for their nomadic lifestyle, though many have settled over time [1]. Approximately 12 million Roma are currently living worldwide and between 7 to 9 million live in Europe [2,3]. Roma are concentrated in Eastern regions of Europe, estimated to comprise 4.9% of the population in Bulgaria and 3.3% of the population in Romania [4,5]. Although the Roma population is the largest minority group in Europe, they often face extreme intolerance and racism [2]. Discrimination towards the Roma population stemmed from the rise of nationalism in the 16th century, but since that time continued oppression and discrimination has been levied on the Roma on the basis of their ethnicity and culture. This has rendered them one of the most socially excluded and marginalized groups in Europe [6].

Family structures within the Roma population tend to be supportive and tight-knit; this structure affords many Roma psychological benefits such as practical support and security, as demonstrated in qualitative studies of Roma families [7,8]. Prosocial behavior, including willingness to share with others, helpfulness and consideration of others feelings is higher among Roma children in comparison to non-Roma children [6]. Though these are positive implications of the Roma family dynamic, negative effects of Roma family life have been documented, including unsafe living conditions and social discrimination [9]. Roma families often dwell in marginalized areas with very little sanitary amenities [10,11]. According to Eurofound, an agency within the European union emphasizing in better living and working conditions, more than half of Roma in Europe did not have access to improved forms of sanitation in their home compared to the majority population [12]. In addition to the Roma population’s living conditions, the Roma children on average have sub-par educational environments. Many Roma children are enrolled in segregated schools or schools designed for the mentally and physical disabled children, which limits their future prospects of educational attainment and employment [3].

With these conditions, the Roma population often is prone to more detrimental health outcomes in comparison to the general population. Studies showed that the Roma have higher risk compared to non-Roma counterparts for animal and human vectored diseases as well as chronic illnesses like cardiovascular disease [13,14]. A study done in the UK found a significantly greater proportion of Roma adults suffers from problems with anxiety and depression than the non-Roma population [2]. Though literature exists for the physical and mental illnesses of the Roma population in general, few studies have examined health among Roma children. From the studies available, Roma children are shown to have higher rates in malnutrition like iron deficiencies and overall cognitive development issues than non-Roma children [13].

The mental health of Roma pre-adolescent children has rarely been studied. The present study draws a study of child mental health among 9,000 European school children, with information on the mental health of more than 100 Roma children in two countries. The aim of this study is to explore the mental health and behavioral disorder of Roma children in comparison to non-Roma children in educational institutions.

## Methods

### Sample

Data was drawn from the School Children Mental Health Project (SCHME), a cross sectional survey designed to estimate the prevalence of and major risk factors in children’s mental health across Europe. Surveys on parents and children were performed in primary schools in seven European countries: Bulgaria, Germany, Italy, Lithuania, Netherlands, Romania and Turkey. School and students within schools were randomly selected for the study. A total of 9021 children participated in the study.

Information on Roma ethnicity was collected in Romania and Bulgaria from which a total of sample of 135 Roma children (in Romania, N = 70; in Bulgaria, N = 65) was obtained. In Romania, Roma ethnic status was obtained via parental report. A total of 70 out of 995 who answered the question identified the child as Roma. In Bulgaria, we identified two schools that were located in areas with almost exclusively Roma populations, thus the children in those schools were identified as Roma children. From the schools in Roma areas, a total of 65 children were sampled. Overall, school participation rates were high in both countries; 95.6% of contacted schools agreed to participate in Romania, and 91.7% in Bulgaria. Parental participation rates were high in Romania (86.6%) and Bulgaria (78.1%), however parental participation was low in the schools in Roma areas (<50%) thus parental reports are not used for analyses of Bulgarian school children. The child participation rate was 67.5% in Romania and 64.1% in Bulgaria. The participation rate of children in the Bulgarian schools identified in Roma areas, 67.7%, was similar compared with the participation rate in remaining schools in Bulgaria (64.1%).

All participating countries had the support of their governments, including their ministers of education and health and received ethical approval from the corresponding authority. This study was approved by the ethics committee of: 1) the Republic of Bulgaria, Deputy Minister of Education, Youth and Science; 2) The German Ministry of Education, Science and Culture, Mecklenburg-Vorpommern; 3) the German State school authority, Luneburg; 4) the German Ministry of Education and Culture of Schleswig-Holstein country; 5) the Italian Ministry of Education and Culture of Schleswig-Holstein country; 6) the Republic of Lithuania – Ministry of Education and Science; 7) the Netherlands Commission of Faculty Ethical Behavior Research (ECG); 8) the Bucharest School Inspectorate General Municipal; and 9) the Istanbul directorate of National Education. Parents received an informational letter and a consent form to be returned to the school. If the parents did not mail to the school a consent form stating their refusal to participate, the child was included. Consent procedures were approved by governing ethical bodies of each country.

### Measures

#### Mental health

Information on the mental health of the children was collected from three informants where available: the parent (we include only mother report in the present study as father reports were rare), the teacher and the child him/herself.

The child report was based on the Dominic Interactive, which assessed symptoms of the following common DSM-IV conditions: Attention Deficit/Hyperactivity Disorder, Conduct Disorder, Oppositional Defiant Disorder, Phobias, Separation Anxiety Disorder, Generalized Anxiety Disorder and Major Depressive Disorder. This instrument is a computerized program which follows an imaginary character, Dominic, through various real-life scenarios and asks the child if he/she has ever empathizes with Dominic in these scenarios. A total of 91 precise situations are used to represent the 7 common DSM-IV childhood pathologies mentioned before. There are pictures, texts and voices to illustrate the abstract emotional and behavioral content of mental health problems and to simply the scenario for a child in primary school. Test-retest reliability and consistency studies done on the Dominic Interactive (and its cross-culture derivatives [15]), showed that this test was reliable (Chronbach’s alpha values in between 0.60 and 0.90 [16-20]). Validity studies suggest that the DI has improved validity compared with other structured interviews of young children [17].

The parents and teacher reports were based on the Strengths and Difficulties Questionnaire (SDQ). The SDQ is a one-page questionnaire for assessing the psychological adjustment of children completed by both parents and teachers [21]. The SDQ asks about 25 attributes which is then divided up into five scales generating scores for emotional symptoms, conduct problems, hyperactivity-inattention, peer problems, and prosocial behavior [21]. A study comparing the Child Behavior Checklist (CBCL) to the SDQ showed that scores from the SDQ and CBCL were highly correlated and equally able to discriminate psychiatric cases [22]. However the SDQ was significantly better than the CBCL at detecting inattention and hyperactivity and the brevity of the SDQ itself made it more preferable than the CBCL among mothers of low-risk children [22].

### Socio-demographics

Age and sex of all children was assessed. The responding parent (which was usually the mother) were additionally were queried regarding maternal and paternal age, education (operationalized as any secondary education versus none) and tobacco consumption (current, occasional, never).

### Statistical analysis

Analyses were done both pooling data from Romania and Bulgaria, as well as separately by country (Bulgaria and Romania) in which Roma children were sampled. First, the distribution of sociodemographic variables obtained from the parental questionnaires was assessed, chi-square tests were used for comparison. In Bulgaria, low completion rates among parents of Roma children were precluded inclusion of parental information in the analysis; in Romania, parental completion rates were high (86.6%). Completion of the Dominic Interactive was high in Roma children across Bulgaria and Romania, thus mental health based on child report by basic demographic characteristics such as age and sex were analyzed in both countries. The SDQ was completed by teachers in both countries; because parental response was low in Bulgaria, we report SDQ results for parents only among those in the Romanian sample.

To assess whether Roma children had increased prevalence of mental health problems odds ratios were estimated from logistic regression, both unadjusted and then adjusted for sex and age of the child. When conducting analyses among the Romanian sample, we also adjusted for factors reported by the parents including mother’s and father’s age, parental smoking and education.

## Results

The sociodemographic characteristics of the samples from both Bulgaria and Romania are presented in Table 1. There was no difference in age or gender in Roma versus non-Roma in either country. In the Romanian sample, Roma parents (both mother and father) were on average younger than non-Roma parents; they were also more likely to smoke and had lower levels of completed education. Specifically, 60.78% of Roma parents had less than a secondary education (high school), compared to 9.22% of non-Roma parents.

**Table 1:** Child sociodemographic factors and other covariates* between Roma and non-Roma in Romania (N = 995) and Bulgaria (N = 1,377)*

		**ROMANIA**			**BULGARIA**		
		**Roma (n =70)**	**Non-Roma (n =925)**	**p-value**	**Roma (n =65)**	**Non-Roma (n =1312)**	**p-value**
		**% (n)**	**% (n)**		**% (n)**	**% (n)**	
**Gender**	Male	48.6% (34)	52.9% (489)	0.4879	53.2% (33)	51.4% (699)	0.7737
	Female	51.4% (36)	47.1% (436)		46.8% (29)	48.6% (662)	
**Average Age**		8.7	8.7	0.6886	9.0	8.8	0.2438
**Mother’s Age**	Under 35	70.8% (46)	54.4% (477)	0.0274	N/A**	N/A**	N/A**
	35-40	15.4% (10)	29.0% (254)				
	Over 40	13.9% (9)	16.7% (146)				
**Father’s Age**	Under 35	60.0% (30)	38.3% (303)	0.0086	N/A**	N/A**	N/A**
	35-40	26.0% (13)	35.9% (284)				
	Over 40	14.0% (7)	25.8% (204)				
**Mother’s smoking Status**	Never	41.0% (25)	72.1% (641)	<0.0001	N/A**	N/A**	N/A**
	Occasional	37.7% (23)	17.2% (153)				
	Current	21.3% (13)	10.7% (95)				
**Mother’s Education**	< Secondary	60.8% (31)	9.2% (76)	<0.0001	N/A**	N/A**	N/A**
	Secondary	33.3% (17)	50.5% (416)				
	> Secondary	5.9% (3)	40.3% (332)				

*Gender and age available on all child respondents; data on parental age and characteristics based on maternal report from the questionnaire data. Insufficient numbers of Roma children parents in Bulgaria completed the questionnaire thus parental data among Bulgarian respondents were not used.

**Due to low parental completion of SDQ, we did not obtain these numbers.

In unadjusted analysis (not shown), Roma children had a higher odds of any internalizing disorder (OR = 3.04, 95% C.I. 2.12 – 4.34), phobias (OR = 4.66, 95% C.I. 3.09–7.03), separation anxiety disorder (OR = 2.53, 95% C.I. 1.72–3.72), generalized anxiety disorder (OR = 3.25, 95% C.I. 1.97–5.36), and major depressive disorder (OR = 3.99, 95% C.I. 2.42–6.56). Further Roma children had a higher odds of any externalizing disorder (OR = 2.81, 95% C.I. 1.82–4.54), oppositional defiant disorder (OR = 3.49, 95% C.I. 2.04–5.99), ADHD (OR = 2.53, 95% C.I. 1.37–4.65), and conduct disorder (OR = 3.43, 95% C.I. 1.95–6.04). We also provide analyses stratified by country in Additional file 1: Table S1 and Additional file 2: Table S2. Results are generally consistent with the pooled analysis, though there were no associations between Roma ethnicity and externalizing disorders in Bulgaria.

Table 2 describes the association between Roma ethnicity and child-reported mental health problems adjusted for sex and age. In adjusted analysis, Roma children had a higher odds of any internalizing disorder (OR = 2.99, 95% C.I. 2.07–4.30), phobias (OR = 4.84, 95% C.I. 3.19–7.35), separation anxiety disorder (OR = 2.54, 95% C.I. 1.72–3.76), generalized anxiety disorder (OR = 2.95, 95% C.I. 1.75–4.96), and major depressive disorder (OR = 3.86, 95% C.I. 2.31–6.37). Further Roma children had a higher odds of any externalizing disorder (OR = 2.84, 95% C.I. 1.78–4.54), oppositional defiant disorder (OR = 3.35, 95% C.I. 1.93–5.82), ADHD (OR = 2.37, 95% C.I. 1.26–4.46), and conduct disorder (OR = 3.63, 95% C.I. 2.04–6.46). We also provide analyses stratified by country in Additional file 1: Table S1 and Additional file 2: Table S2. Results are generally consistent with the pooled analysis, though there were no associations between Roma ethnicity and externalizing disorders in Bulgaria.

**Table 2:** **Prevalence and odds of child-reported psychiatric disorders between Roma and non-Roma children in Romania and Bulgaria (N = 2,372)**

	**Proportion of Roma (n =135)**	**Proportion of Non-Roma (n =2237)**	Adjusted* Odds Ratio (95% CI)
**Internalizing Disorders**	47.7% (62)	23.1% (499)	2.99 (2.07, 4.30)
**Phobia**	28.5% (37)	7.87% (170)	4.84 (3.19, 7.35)
**Separation Anxiety Disorder**	32.3% (42)	15.9% (343)	2.54 (1.72, 3.76)
**General Anxiety Disorder**	16.2% (21)	5.6% (121)	2.95 (1.75, 4.96)
**Major Depressive Disorder**	16.9% (22)	4.9% (105)	3.86 (2.31, 6.37)
**Externalizing Disorders**	20.0% (26)	8.0% (173)	2.84 (1.78, 4.54)
**Oppositional Defiant Disorder**	13.9% (18)	4.4% (95)	3.35 (1.93, 5.82)
**Attention Deficit Hyperactive Disorder**	10.0% (13)	4.2% (91)	2.37 (1.26, 4.46)
**Conduct Disorder**	12.3% (16)	3.9% (85)	3.63 (2.04, 6.46)

*Adjusted for sex and age of the child.

We also examined differences in mental health between Roma and non-Roma based on teacher report. In unadjusted analysis (not shown), teachers reported that Roma children had a higher prevalence of all reported disorders (emotional, conduct disorder, hyperactivity, peer-relational problems and prosocial).

Table 3 shows the association between Roma ethnicity and teacher report of mental health problems adjusted for child’s age and sex. In adjusted analysis, Roma children had higher odds of emotional problems (OR = 2.03, 95% C.I. 1.20-3.44), peer-relational problems (OR = 2.76, 95% C.I. 1.73-4.41) and prosocial behavior (OR = 2.75, 95% C.I. 1.75-4.33). We also provide analyses stratified by country in Additional file 3: Table S3. Results were generally consistent with those reported in Table 3, though the magnitude of the association between Roma ethnicity and emotional problems was lower in Bulgaria (OR = 1.38, 95% C.I. 0.53-3.60) than in Romania (OR-3.81, 95% C.I. 1.58-9.21). Further, there were no associations between Roma ethnicity and parental report of psychopathology in Romania.

**Table 3:** **Odds of teacher-reported psychiatric disorders between Roma and non-Roma children in Romania and Bulgaria (N = 2103)**

	**SDQ (Teachers)**
	Adjusted OR* (95% CI)
**Emotional problems**	2.03 (1.20, 3.44)
**Conduct problems**	1.48 (0.94, 2.35)
**Hyperactivity**	1.55 (0.96, 2.51)
**Peer Relation problems**	2.76 (1.73, 4.41)
**Prosocial problems**	2.75 (1.75, 4.33)

*Adjusted for sex and age of the child.

## Discussion

This is the first study of mental health outcomes among preadolescent Roma children. In a community based sample of Roma and non-Roma children across Romania and Bulgaria, the present study reports that Roma children have a higher risk of internalizing mental disorders than non-Roma children in both Bulgaria and Romania. Higher reports of externalizing disorders among Roma children are also observed among Roma children in Romania. These increased rates of mental health problems were confirmed by teacher report.

We note that Roma children and their teachers reported higher levels of mental health problems, whereas few differences between Roma and non-Roma were evident based on parent report in Romania. The differences observed by the informants may be explained by various social factors. Because the Roma face considerable discrimination, parents may conceptualize mental illness symptoms as potentially stigmatizing and thus have a greater inclination to underreport the mental illness of their child [23,24]. This coincides with census underreporting by Roma for the fear of eviction [11,25]. Teachers on the other hand, due to their possible discrimination against this group thus more attention towards their behaviors, may over-report the mental illness of children. Qualitative reports from Roma communities stated that teachers often favor non-Roma children over Roma children [25]. For example, according to various recent reports, a Roma child was violently attacked by 8 other children, and the teacher supported the non-Roma children [26]. Other teachers may have low expectations from minority groups and often have a pre-conceived notion and discrimination about their cultural upbringing [27]. Roma children are often systematically misdiagnosed of mental disability and are thus often placed in special classes meant for mentally handicapped children [26].

We also observed that there was a difference in how certain disorders were reported depending on the environment Roma children are exposed to. Roma teachers in Romania reported an increased odds of internalizing disorders compared to non-Roma children while in Bulgaria, teachers reported higher odds of externalizing disorders (with the exception of hyperactivity). We also observed that Roma children in Romania reported increased odds of externalizing disorders compared non-Roma children, whereas in Bulgaria there was no increased odds of externalizing disorders. We note that in Bulgaria, the sampled Roma children were ascertained from Roma-only schools, whereas in Romania the Roma children were interspersed with non-Roma children. The teacher’s potentially biased perception of the Roma population could account for the increased perception of externalizing disorder among the Roma population in Bulgaria as well as the internalizing disorder in Romania. For children however, in an environment of other Roma peers, acting out or other externalizing behaviors could be more normative, and there could be less variance overall attributable to externalizing behaviors among children sampled from within the school environment. Experiences of discrimination may engender identification and association within ethnic group [28], as evidenced by development of ethnic enclaves that have potentially positive mental health benefits among other marginalized groups [28,29]. In a study looking at mental health among adult refugees residing in Denmark, they found a significant association between perceptions of discrimination and internalizing behavior, but not for externalizing behavior [28]. Thus, children sampled in Romania may be subjected to discrimination by non-Roma peers, whereas children in Bulgaria may experience less discrimination.

There are several limitations of this study that could be expanded with future study. First, it was difficult to obtain parental responses to the questionnaire among the Roma parents in Bulgaria. Thus, we did not have information on potentially important covariates such as parental age, smoking status and education for Bulgarian Roma. However, we had high parental participation rates among Roma children in Romania, providing evidence of a robust association between Roma ethnicity and mental health factors that is independent of sociodemographic characteristics. Further, clear identification of Roma children and parents in the community is a challenge; many parents do not report Roma ethnic status due to fear of stigma and discrimination; thus we may be undercounting the number of Roma children in our sample, especially in Romania where children were not segregated based on ethnic group. Further, there may have been Roma children in Bulgarian schools that were not located in Roma areas. Due to the sensitivity of identifying Roma ethnic status, we did not include a question on Roma ethnicity on the Bulgarian questionnaire. Thus, the children identified as “Roma” in our sample are known Roma ethnicity, though there may be Roma children in the remaining sample. Results may be conservative estimates of associations, assuming that Roma children misclassified as non-Roma have similar mental health risks as identified Roma children. Further, historically a lower proportion of Roma children enroll in primary school compared with non-Roma children [30], thus the children in our sample may comprise a select subset of Roma children. However, the introduction of Bulgaria and Romania into the European Union in 2007 portended compliance with European compulsory schooling policy, thus the under-representation of Roma children in formal education is likely shifting. Nevertheless, the Roma children who are not in formal school may differ than those who attend formal school; we would expect higher mental health burden among non-school attending Roma children, thus our estimates are again likely conservative.

The Roma population faces many hardships as minorities in Europe and has higher rates of adverse health outcomes compared with majority population. In Serbia, for example, the mortality rate is much higher in the Roma population than in the non-Roma population with cardiovascular disease, neoplasm and respiratory illnesses being the most common among [31]. The age structure of this population in Serbia resembles those of developing countries with a high infant population and low elderly population, a state much different from the rest of the Serbian population. Birth defects have been established to be worse among Roma children [32], yet other non-communicable diseases like mental illnesses have not been established. Our findings indicate that mental health care access is needed for young Roma children, especially given that early onset of adverse mental health predicts increased problems throughout the life course [33,34]. There are current programs in the United States that target the mental health of children in poor-income families and minorities which often involve social support [35]. Studies have also shown that cognitive behavioral therapy (CBT) [36] as well as clinical case management as a supplement to CBT [37] seem to be effective with ethnic minorities. The effectiveness of these interventions on the Roma population should also be examined. In addition an advocacy for health among the Roma population should be initiated to allow for the Roma population to be more active about the knowledge of their health. Many Roma families often ignore health problems due to their social seclusion from the general population [13]. An effort to promote health literacy among the Roma population should be initiated. These efforts should be culturally sensitive and done in a way to empower this population rather than further seclude them.

## Conclusion

The present study documents mental health disparities among Roma compared with non-Roman children in Bulgaria and Romania. In both countries, Roma children have higher rates of internalizing disorders than non-Roma, and in Romania, we document higher rates of externalizing disorders as well. Given the lifecourse associations between onset of mental health problems in childhood and further mental as well as physical disorders persisting throughout adulthood, these results suggest that addressing mental health disparities in childhood could be an important strategy to improve population health for the Roma. Effective and low-cost interventions are well established, and resources to embed these strategies in Roma communities are critical for population health in Europe.

### Key messages

There are stark mental health disparities between Roma children and their non-Roma peers.

Roma children have between two and six times higher odds of internalizing disorders compared with non-Roma peers, and Roma children intregrated in schools with non-Roma have higher odds of externalizing disorders.

Stigma and discrimination, poor living conditions, and segregation may adversely impact the health of Roma children.

## Additional files

Additional file 1: Table S1. Prevalence and odds of child-reported psychiatric disorders between Roma and non-Roma children in Romania (N = 995). Additional file 2: Table S2. Prevalence and odds of child-reported mental health problems between Roma and non-Roma children in Bulgaria (N = 1,377). Additional file 3: Table S3. Odds of parent- and teacher-reported psychiatric disorders between Roma and non-Roma children.
