# Supplementary material for: Mental health disparities between Roma and non-Roma children in Romania and Bulgaria
Source: BMC Psychiatry. 2014 Nov 18;14:297. doi: 10.1186/s12888-014-0297-5 (PMC4240804; doi:10.1186/s12888-014-0297-5)
Supplement: Additional file 1: Table S1. — Prevalence and odds of child-reported psychiatric disorders between Roma and non-Roma children in Romania (N = 995). [file 12888_2014_297_MOESM1_ESM.doc]

| **Online Table S1: Prevalence and odds of child-reported psychiatric disorders between Roma and non-Roma children in Romania (N=995)** | | | |
| --- | --- | --- | --- |
|  | **Proportion of Roma (n = 70)** | **Proportion of Non-Roma (n = 925)** | **Adjusted* Odds Ratio (95% CI)** |
|  |  |  |  |
| **Internalizing Disorders** | 42.7% (29) | 21.2% (191) | 2.67 (1.22, 5.84) |
| **Phobia** | 27.9% (19) | 8.8% (79) | 3.35 (1.35, 8.30) |
| **Separation Anxiety Disorder** | 26.5% (18) | 14.6% (132) | 2.08 (0.89, 4.88) |
| **General Anxiety Disorder** | 14.7% (10) | 3.0% (27) | 5.26 (1.63, 17.05) |
| **Major Depressive Disorder** | 13.2% (9) | 3.0% (27) | 2.74 (0.76, 9.95) |
|  |  |  |  |
| **Externalizing Disorders** | 25.0% (17) | 4.8% (43) | 9.85 (3.74, 25.95) |
| **Oppositional Defiant Disorder** | 19.1% (13) | 2.6% (23) | 9.53 (3.00, 30.33) |
| **Attention Deficit Hyperactive Disorder** | 11.8% (8) | 2.0% (18) | 10.70 (3.13, 36.60) |
| **Conduct Disorder** | 17.7% (12) | 3.1% (28) | 9.93 (3.27, 30.17) |
| * Adjusted for sex, age, mother's age, father's age, smoking status and education | | | |
