# Supplementary material for: Mental health disparities between Roma and non-Roma children in Romania and Bulgaria
Source: BMC Psychiatry. 2014 Nov 18;14:297. doi: 10.1186/s12888-014-0297-5 (PMC4240804; doi:10.1186/s12888-014-0297-5)
Supplement: Additional file 2: Table S2. — Prevalence and odds of child-reported mental health problems between Roma and non-Roma children in Bulgaria (N = 1,377). [file 12888_2014_297_MOESM2_ESM.doc]

| **Online Table S2: Prevalence and odds of child-reported mental health problems between Roma and non-Roma children in Bulgaria (N=1,377)** | | | |
| --- | --- | --- | --- |
|  | **Proportion of Roma (n = 65)** | **Proportion of Non-Roma (n = 1312)** | **Adjusted* Odds Ratio (95% CI)** |
|  |  |  |  |
| **Internalizing Disorders** | 53.2% (33) | 24.5% (308) | 3.57 (2.10, 6.07) |
| **Phobia** | 29.0% (18) | 7.2% (91) | 6.08 (3.21, 10.83) |
| **Separation Anxiety Disorder** | 38.7% (24) | 16.8% (211) | 3.34 (1.93, 5.78) |
| **General Anxiety Disorder** | 17.7% (11) | 7.5% (94) | 2.23 (1.06, 4.69) |
| **Major Depressive Disorder** | 21.0% (13) | 6.2% (78) | 3.84 (1.95, 7.56) |
|  |  |  |  |
| **Externalizing Disorders** | 14.5% (9) | 10.3% (130) | 1.29 (0.60, 2.79) |
| **Oppositional Defiant Disorder** | 8.1% (5) | 5.7% (72) | 1.14 (0.40, 3.23) |
| **Attention Deficit Hyperactive Disorder** | 8.1% (5) | 5.8% (73) | 1.11 (0.39, 3.16) |
| **Conduct Disorder** | 6.5% (4) | 4.5% (57) | 1.48 (0.51, 4.25) |
| *Adjusted for sex and age of the child | | | |
|  |  |  |  |
