# Supplementary material for: Mental health disparities between Roma and non-Roma children in Romania and Bulgaria
Source: BMC Psychiatry. 2014 Nov 18;14:297. doi: 10.1186/s12888-014-0297-5 (PMC4240804; doi:10.1186/s12888-014-0297-5)
Supplement: Additional file 3: Table S3. — Odds of parent- and teacher-reported psychiatric disorders between Roma and non-Roma children. [file 12888_2014_297_MOESM3_ESM.doc]

| **Online Table S3: Odds of parent- and teacher-reported psychiatric disorders between Roma and non-Roma children** | | | |
| --- | --- | --- | --- |
|  | **ROMANIA** | | **BULGARIA** |
|  | **SDQ (Parents)** | **SDQ (Teachers)** | **SDQ (Teachers)** |
|  | **OR (95% CI)** | **OR (95% CI)** | **OR (95% CI)** |
| **Emotional** | 0.81 (0.36, 1.81) | 3.81 (1.58, 9.21) | 1.38 (0.53, 3.60) |
| **Conduct** | 1.47 (0.68, 3.21) | 1.41 (0.61, 3.26) | 1.86 (0.98, 3.53) |
| **Hyperactivity** | 0.61 (0.22, 1.68) | 1.84 (0.79, 4.32) | 1.36 (0.69, 2.71) |
| **Peer Relations** | 1.21 (0.59, 2.48) | 2.55 (0.92, 7.08) | 3.23 (1.74, 6.02) |
| **Prosocial** | 2.24 (0.52, 9.57) | 2.71 (0.97, 7.55) | 3.09 (1.71, 5.58) |
| In Romania, adjusted for child’s age and sex, parental smoking and education. In Bulgaria, adjusted for child’s age and sex. | | | |
